# Dysbiosis and colorectal cancer: conducive factors, biological and molecular role, and therapeutic prospectives

**DOI:** 10.37349/etat.2025.1002329

**Published:** 2025-06-27

**Authors:** Gurkaranjot Singh, Zuhair Chaudhry, Anik Boyadzhyan, Kayvan Sasaninia, Vikrant Rai

**Affiliations:** University of Barcelona, Spain; ^1^College of Osteopathic Medicine of the Pacific, Western University of Health Sciences, Pomona, CA 91766, USA; ^2^Department of Translational Research, Western University of Health Sciences, Pomona, CA 91766, USA

**Keywords:** Colorectal cancer, intestinal microbiome, inflammation, dysbiosis, immunomodulation, therapeutics

## Abstract

Colorectal cancer (CRC) is the third leading cause of cancer-related death in the United States. Emerging evidence highlights the significant role of gut microbiota dysbiosis, characterized by a reduction in beneficial bacteria and an increase in pro-inflammatory and pro-carcinogenic bacteria, in CRC pathogenesis. Both genetic and environmental factors, including diet, antibiotic use, physical activity, aging, and obesity, contribute to this microbial imbalance. Dysbiosis promotes chronic inflammation and immune dysregulation, which facilitates tumor initiation and progression. This review examines the intricate interactions between gut microbiota, immune modulation, and CRC development. It explores current and emerging therapeutic strategies that target the microbiome to enhance treatment efficacy, discusses interventions aimed at restoring healthy microbiota in CRC patients, and outlines future directions for microbiome-based therapies to improve clinical outcomes.

## Introduction

Colorectal cancer (CRC) imposes a great deal on global health; it is the third most frequent cancer diagnosis and the third leading cause of cancer-related deaths in both genders in the US. The second most common killer among all cancer-related deaths is CRC, and in men under 50 years of age, it is the leading cause [1]. Men are approximately 1.5 times more likely than women to develop CRC, regardless of age or ethnicity [2]. African Americans tend to have an earlier onset, higher mortality, and higher incidence [3]. The survival rate for patients with localized cancer is 90% at 5 years, and for patients across all stages of CRC combined is 63% at 5 years [4]. However, metastatic CRC still proves to be lethal, as the 5-year survival rate is around 14% [5]. CRC in the colon or rectum results from the growth of the glandular epithelial cells inside the colon or the rectum [6].

Individuals with an increased risk of CRC include those who have had cancer, a history of colon polyps, inflammatory bowel disease (IBD), diabetes mellitus, or cholecystectomy. Lifestyle factors, including overweight and obesity, physical inactivity, cigarette smoking, alcohol consumption, a diet low in fiber, fruits, vegetables, calcium, and dietary products, and a diet high in red and processed meat, increase CRC risk. In addition, gut microbiome, age, gender, race, and socioeconomic status are known to influence the risk of CRC [7]. Typical symptoms of CRC include rectal bleeding, iron deficiency anemia, weight loss, fatigue, and/or abdominal/rectal pain [4, 8]. CRC develops either sporadically, which is the vast majority of cases, or heritably, which makes up 10% of cases [9]. Because of its genetic pathogenesis, the prevention of CRC is difficult, and that is why screening plays an important role in fighting cancer. Stool-based tests, computed tomography colonography, and sigmoidoscopy are the main methods to diagnose CRC. Stool-based tests include the guaiac fecal occult blood test (gFOBT), detecting blood in stool via pseudoperoxidase activity of heme, and the fecal immunochemical test (FIT) using antibodies to detect human globin. Sigmoidoscopy provides direct visualization of the colorectum and the opportunity to biopsy and/or remove polyps [10]. Sigmoidoscopy has led to a notable decrease of 20% in the incidence of CRC [11]. Despite these screening modalities, the prevalence of CRC is increasing. Thus, there is a need to understand the underlying molecular mechanisms, novel therapeutic targets, and diagnostic methods for screening, early diagnosis, and developing better treatment modalities.

## Molecular mechanisms and gut microbiota

CRC develops on a background of IBD, which includes Crohn’s disease (CD) affecting the entire gastrointestinal (GI) tract and ulcerative colitis (UC) affecting mainly the colon and rectum. The significantly increased risk of developing CRC in IBD patients is largely attributed to the chronic inflammation associated with IBD, which can lead to dysplastic changes that can eventually progress to cancer. CRC is due to the combined effects of genetic and environmental influences on the body (discussed above). At the cellular level, chromosomal instability, characterized by a genetic abnormality that includes number alterations, indels, translocations, amplifications, and loss of heterozygosity, is responsible for 65–70% of CRC development. Chromosomal instability arises because of dysfunctional chromosomal segregation, disordered telomere viability, and ineffective DNA-damage response machinery [12]. For example, somatic mutation of the tumor suppressor gene *FBXW7* (F-box and WD repeat domain containing 7) accounts for nearly 16% of CRC patients. *FBXW7* mutation is associated with a higher tumor mutation burden, a higher microsatellite instability (MSI) score, and a lower chromosomal instability score. MSI is responsible for 15% of CRC cases, which is due to mismatch repair errors [12].

*FBXW7* mutation with a higher MSI score results in a higher infiltration of M1 macrophage, CD8+ T cell, and regulatory T (Treg) cell and significant enrichment of interleukin (IL)-6 Janus kinase/signal transducer and activator of transcription 3 (JAK/STAT3) signaling, p53 pathway, and interferon (IFN)-γ and IFN-α response in CRC patients [13]. *FBXW7β*, an isoform of *FBXW7*, is an E3 ligase of fatty acid synthase (FASN) that, when mutated, leads to lipogenesis [14]. The increase of FASN regulates CRC metastasis by assisting in its growth and survival. FASN converts carbohydrates into fatty acids that are then converted into lipids that can either be stored or used as needed [15]. This allows the tumor to maintain its energy homeostasis and continue growing. Serrated neoplasms (a type of polyp in the colon, if left untreated, can develop into bowel cancer) make up 15–25% of CRCs, which typically arise from a *BRAF* gene activation, resulting in methylation of CpG islands [9, 12].

**Figure 1:**
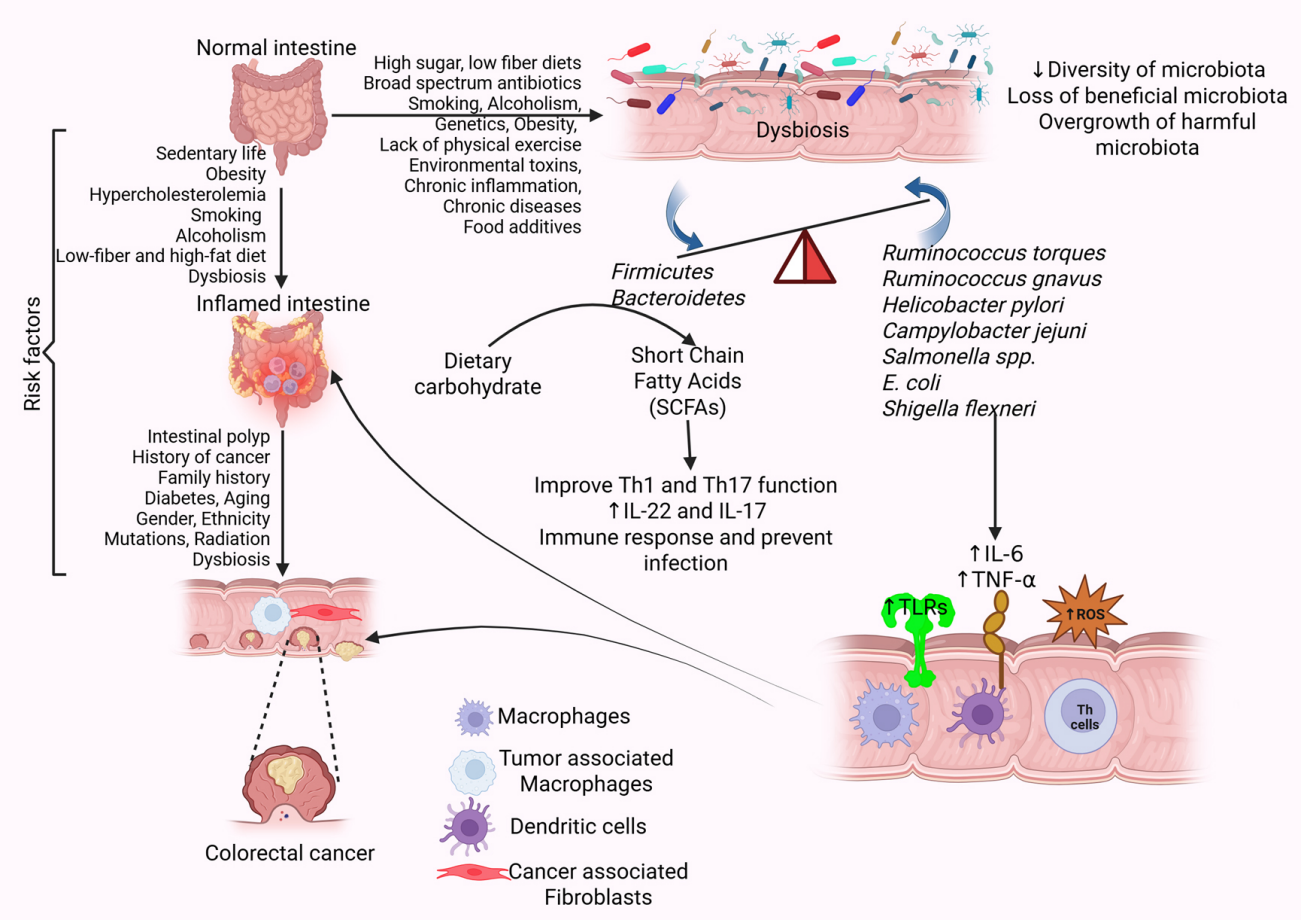
**Risk factors and pathophysiology of CRC.** Gut microbiota dysbiosis, influenced by environmental and lifestyle factors, creates a favorable environment for CRC. The presence of risk factors, including diabetes, smoking, alcoholism, chronic inflammation, and others, leads to the alteration of the intestinal microbiota termed dysbiosis. Gut dysbiosis increases the secretion of proinflammatory cytokines such as IL-6 and TNF-α, expression of TLRs, and oxidative stress by increasing the production of ROS. This disrupts the intestinal barrier and increases the propensity for CRC. Further, the presence of polyps in the intestine also increases the risk of CRC. CRC: colorectal cancer; IL: interleukin; ROS: reactive oxygen species; TLRs: toll-like receptors; TNF: tumor necrosis factor. Created in BioRender. Rai, V. (2025) https://BioRender.com/it0zune

Gut microbiota research in recent years has gained traction, especially in its association with CRC. The gut microbiome is considered very important as a mediator for the pathogenesis of CRC, working as a dynamic ecosystem at the interface of genetic and environmental factors between the host. Gut microbiota is a complex community consisting of trillions of microorganisms, including bacteria, fungi, and protists, living in a symbiotic relationship with humans. They play a critical role in nutrition, protection, and digestion [16]. Though gut microbiota varies in each person, phyla *Firmicutes* and *Bacteroidetes* make up to 90% of the gut microbiome. The gut microbiota gets its nutrients from dietary carbohydrates, fermenting them to create short-chain fatty acids (SCFAs) [17]. SCFAs are known to boost the functions of Th1 and Th17 cells, which prevent and fight infection. SCFAs also increase the production of the effector cytokines IL-22 and IL-17 during active immune responses, which are key to preventing infection and fighting pathogens [18] (Figure 1). However, disruption in microbiota with a decrease in beneficial and an increase in harmful microbes, called dysbiosis, is due to a decrease in the diversity of microbiota and can cause negative effects. Dysbiosis can be caused by various factors such as genetic background, health status (infections, inflammation), and lifestyle habits. Environmental factors such as diet (high sugar, low fiber), antibiotics, drugs, food additives, and hygiene have also been shown to cause dysbiosis [19].

Specific bacterial species, such as *Fusobacterium nucleatum* (*F. nucleatum*), *Escherichia coli* (*E. coli*), and *Bacteroides fragilis* (*B. fragilis*), carry genotoxins and metabolites that participate in DNA damage, activation of oncogenic pathways, and suppression of anti-tumor immune mechanisms. Also, microbiota metabolites such as SCFAs have protective effects, and secondary bile acids (SBAs) are potentially carcinogenic over the direct metabolism of primary bile acids in bacteria. This can cause IBD, obesity, diabetes mellitus, and CRC [17, 20] (Figure 1). Dysbiosis creates a microenvironment, perfect for CRC to develop. As previous studies have found a relationship between dysbiosis and CRC, not much has been utilized in the treatment and prevention of CRC. This review aims to comprehensively discuss the role of gut microbiota in CRC and potential therapeutic aspects.

## Intestinal immunity and microbiota

The GI tract serves as the interactive framework for both the gut microbiota and the gut immune system. The initial layer of the gut immune system, which includes gut-associated lymphoid tissue and Peyer’s patches, develops through essential interactions with gut commensals. This established immune barrier and its associated mechanisms restrict direct contact between commensals and the epithelial cell surface. The second layer of immunity swiftly detects and eliminates bacteria without allowing their entry into intestinal tissues. The third tier of immune responses operates within the mucosa, inhibiting the activation of the systemic immune system. The innate immune barrier consists of mucus, antimicrobial peptides (AMPs), and secretory IgA (sIgA) [21]. Chronic inflammation of the GI tract is one of the highest risk factors for developing CRC. Chronic inflammation and epithelial damage can promote DNA damage, dysplasia, and ultimately CRC. To understand how disruptions in host intestinal immunity may contribute to colorectal carcinogenesis, it is essential to examine the key components of the innate immune barrier. These include the mucus layer, AMPs, sIgA, and various innate immune cells, each plays a distinct role in maintaining intestinal homeostasis and preventing chronic inflammation. The following subsections will explore how perturbations in the host’s innate immunity contribute to the development of CRC.

### Mucus layer

**Figure 2:**
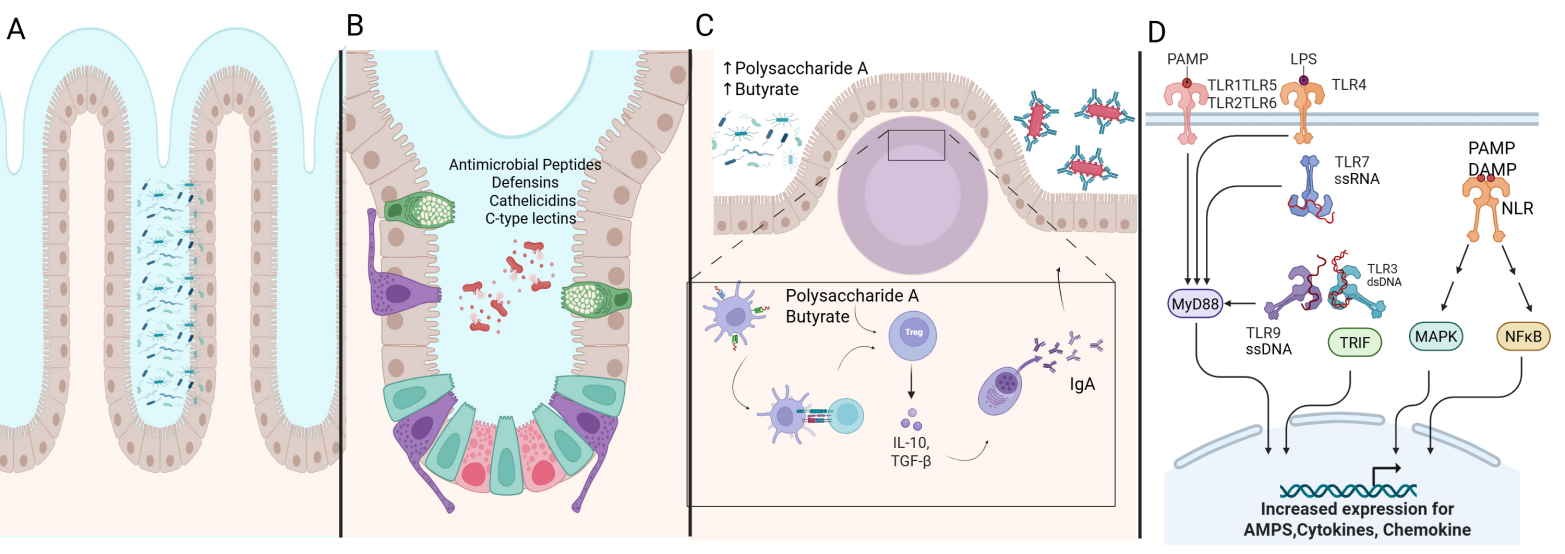
**Intestinal immunity and microbiota in gut health.** (**A**) The mucus layer forms the first line of defense in the GI tract to prevent microbial attachment to epithelial cells. (**B**) Antimicrobial peptides, including defensins, cathelicidins, and C-type lectins, secreted by Paneth cells and other epithelial cells, inhibit microbes. (**C**) Secretory IgA traps pathogens and toxins in the mucus. Polysaccharide A and butyrate from commensal bacteria promote Treg-mediated anti-inflammatory responses. (**D**) Pattern recognition receptors like TLRs and NLRs detect microbial-associated molecular patterns. This triggers the immune response pathway involving MyD88, TRIF, MAPK, and NFκB to protect intestinal cells. Activation of these pathways regulates immune responses and inflammation, and the acute immune response protects the intestinal epithelium. However, in the presence of chronic inflammation, this regulation is lost and results in IBD. AMPs: antimicrobial peptides; DAMP: damage-associated molecular pattern; GI: gastrointestinal; IBD: inflammatory bowel disease; IL: interleukin; LPS: lipopolysaccharide; MAPK: mitogen-activated protein kinases; MyD88: myeloid differentiation primary response 88; NLR: NOD-like receptor; PAMP: pathogen-associated molecular pattern; TGF-β: transforming growth factor beta; TLR: Toll-like receptor; Treg: regulatory T; TRIF: TIR-domain-containing adapter-inducing interferon-β. Created in BioRender. Rai, V. (2025) https://BioRender.com/e7t5oem

The mucus layer in the GI tract, composed of mucin glycoproteins from goblet cells, forms a protective, gel-like barrier on the intestinal epithelia, preventing microbial attachment and facilitating luminal content transport (Figure 2A). This layer consists of a peptide backbone with glycosylated and non-glycosylated domains, primarily featuring oligosaccharides like *N*-acetylglucosamine, *N*-acetylgalactosamine, fucose, and galactose, terminating in sialic acid or sulfate groups [22]. Besides serving as a barrier, mucins provide carbohydrates and peptides for commensals such as *Bacteroides thetaiotaomicron* and *B. fragilis* [23] and *Bifidobacterium bifidum* [24]. Mucolytic bacteria like *Ruminococcus torques* and *Ruminococcus gnavus* have been demonstrated to degrade mucin in vitro and enhance other nonmucolytic bacteria to utilize mucin as a nutrient source [25]. The degradation of mucin potentially contributes to the thinning of the mucus layer. Pathogens like *Helicobacter pylori*, *Campylobacter jejuni*, *Salmonella* spp., *E. coli*, and *Shigella flexneri* have evolved mechanisms to breach the mucus barrier, including urease production, flagella, and mucin-binding proteases [26, 27]. The depletion of the mucus layer, in addition to mechanisms that breach the mucus barrier, increases microbial-epithelial contact, promoting inflammation and mucosal damage, which can lead to symptoms characteristic of irritable bowel disease. Clinical manifestations of these events may involve persistent changes in bowel habits, rectal bleeding, or abdominal discomfort mimicking irritable bowel syndrome [26, 27].

### Antimicrobial peptides

AMPs such as defensins, cathelicidins, and C-type lectins are secreted by intestinal epithelial cells, including enterocytes, Goblet, and Paneth cells, to restrict microbial access to epithelia (Figure 2B). These small cationic proteins (20–40 amino acids) bind to negatively charged microbial membranes or enzymatically disrupt the cell wall. Defensins are classified into α, β, and θ types. α-Defensin, produced as pro-cryptdin and converted to mature-cryptdin by matrix metalloproteinase (MMP)-7, is essential for microbial defense [28]. Mice lacking MMP-7 cannot produce mature cryptdin and are highly susceptible to *Salmonella typhimurium* [29]. β-Defensin is expressed in small and large intestinal epithelia via nucleotide-binding oligomerization domain 2 (NOD2) signaling, with *Nod2*-deficient mice showing increased susceptibility to *Listeria monocytogenes* [30]. θ-Defensin is found only in rhesus macaques’ leukocytes. Cathelicidin-related antimicrobial peptide (mCRAMP), encoded by *Cnlp*, is restricted to colonic epithelia, with *Cnlp*–/– mice exhibiting increased *Citrobacter rodentium* adherence [31]. C-type lectins, with over 1,000 proteins, play a critical role in innate and adaptive immunity. For instance, RegIIIγ, a secreted antibacterial lectin, creates a ~50 μm zone separating microbiota from the small intestine epithelial surface [32].

Disruption of AMP expression has been associated with the development of CRC. An analysis of four independent CRC patient cohorts demonstrated a significant and consistent decrease in human β-Defensin 1 (HBD1) expression in CRC tissues. EGFR (epidermal growth factor receptor) signaling, a pathway often upregulated in CRC, represses HBD1 when activated in colon tumor tissue cell lines [33]. Human α defensin 5 (DEFA5) and DEFA6 dysregulation are also associated with CRC. DEFA5 exhibits an inhibitory effect on colon cancer cell lines, whereas DEFA6 overexpression was associated with tumorigenesis [34]. An analysis of 650 CRC tissue samples and 50 paired normal colorectal mucosa samples revealed that the colorectal tissue showed a significant reduction in expression of cathelicidin (LL-37). These findings suggest that the disruption of AMP expression contributes to CRC pathogenesis through impaired mucosal barrier function and modulation of oncogenic signaling pathways. Altered expression of AMPs may facilitate microbial dysbiosis and sustained inflammation, creating a microenvironment conducive to tumor initiation and progression. Moreover, the differential roles of specific defensins (e.g., tumor-suppressive DEFA5 vs. tumor-promoting DEFA6) highlight the complexity of AMP regulation in CRC and suggest that targeted modulation of AMP pathways could represent a novel therapeutic strategy for CRC [35].

### IgA

sIgA, a crucial defense mechanism, protects intestinal epithelia from enteric toxins and pathogens while maintaining immune tolerance (Figure 2C). In Peyer’s patches, macrophage-like C-X3-C motif receptor 1 (CX3CR1)+ dendritic cells (DCs) capture luminal antigens and transfer them to migratory CD103+ DCs, which then interact with Treg cells to induce B cells to produce IgA, aided by retinoic acid, transforming growth factor beta (TGF-β), IL-10, and thymic stromal lymphopoietin [36, 37]. The IgA produced by plasma B cells translocates to the intestinal lamina propria and across the epithelial layer, where it entraps bacteria in mucus, neutralizing toxins and pathogens, and facilitating their removal by peristaltic and mucociliary actions [38]. The balance between commensals and pathobionts influences IgA secretion, with commensal-derived polysaccharide A from *B. fragilis* and metabolites like butyrate inducing anti-inflammatory responses via Treg cells. In dysbiosis, certain commensals act as pathobionts, triggering pro-inflammatory responses, as seen in experimental colitis, where *H. hepaticus* activates TH17 cells through IL-17, IL-23, and tumor necrosis factor (TNF)-α. Malnourishment can alter the gut microbial composition and IgA responses, as demonstrated by the transfer of a malnourished infant’s IgA(+) bacterial consortium to gnotobiotic mice, which led to epithelial barrier disruption, weight loss, and sepsis, conditions preventable by administering IgA-targeted bacteria from healthy microbiota [39].

Several studies demonstrate that disruptions to IgA regulation can contribute to the development of CRC. An assessment of colon tissue from adenocarcinoma patients revealed a significant reduction in IgA-secreting plasma cells due to impaired chemokine homing signals to the mucosal tissue [40]. *Igha* knockout mice (*Igha*–/–) were observed to have increased spontaneous inflammation localized to the ileum with an increase in CD4+ T cells, elevated IFN-g and IL-17, increased ileal segmented filamentous bacteria, and perturbed gut microbiota composition [41]. In a CRC mouse model, deletion of marginal zone and B1 cell-specific protein (*Mzb1*–/–), resulting in IgA deficiency, demonstrated an increase in tumor nodule size and number compared to wild-type controls. Oral supplementation of monoclonal IgA in *Mzb1*–/– mice alleviated inflammation and CRC progression [42]. Collectively, these studies suggest IgA deficiency is associated with increased intestinal inflammation, microbial dysbiosis, and increased susceptibility to CRC, with potential avenues of IgA monoclonal antibody therapy in CRC.

### Innate and adaptive immunity

The innate immune response distinguishes between commensals and pathogens through germline-encoded pattern-recognition receptors (Figure 2D). Toll-like receptors (TLRs) identify conserved microbial-associated molecular patterns such as bacterial lipopolysaccharide (LPS), lipoproteins, flagellin, and unmethylated CpG DNA. TLR subfamilies have specific targets: TLR1, TLR2, and TLR6 recognize lipids, while TLR7, TLR8, and TLR9 recognize nucleic acids; TLR5 identifies flagellin, and TLR4 can recognize diverse ligands like LPS and paclitaxel [43]. Cytosolic antigens, including γ-*D*-glutamyl-meso-diaminopimelic acid and muramyl dipeptide, are recognized by NOD-like receptors [44, 45]. Antigen-presenting cells like DCs and macrophages express TLRs, ingest and degrade pathogens, and produce co-stimulatory molecules and cytokines. These cytokines aid in differentiating naive CD4+ T cells into various subsets, including T helper 1 (TH1), TH2, TH17, Treg, and TR1 cells. A balance between Treg cells and CD4+ effector T cells in the intestinal mucosa helps discriminate between commensal and pathogenic microbes. Microbial dysbiosis can trigger an inflammatory response mediated by TH1, TH2, and TH17 cells. TH1 and TH2 activation is marked by pro-inflammatory cytokines like IL-4, IL-5, IFN-γ, and IL-13, while TH17 cells produce IL-17, which stimulates stromal cells to express pro-inflammatory cytokines such as IL-6, IL-8, and IL-22 [46, 47].

TLRs and NOD-like receptors have been reported to be associated with modulating CRC risk. Strong upregulation of TLR4 in colonic tissues has been associated with the progression of UC and CRC [48]. Deletion of *TLR4* protected *TLR4*–/– mice from tumor progression [49]. Similarly, the inhibition of TLR4 signaling pathways also attenuated intestinal inflammation and impeded tumorigenesis in the colon of BALB/c mice [50]. TLR9 was also found to be upregulated in a CRC mouse model [51]. Inhibiting TLR9 in the CRC cell line HT29 decreased the cell viability and proliferation of CRC cells. Polymorphisms in TLR2, 3, 4, 5, and 9 have been reported to modulate CRC risk and development in humans. *Nod2* deficiency in *Nod2*–/– mice significantly enhanced inflammatory pathways and epithelial cell proliferation in hyperplastic regions, indicating *Nod2*-deficient mice are highly susceptible to CRC [51–53]. Together, these findings indicate dysregulation of TLR and NOD-like receptor signaling can disrupt intestinal homeostasis and inflammation-induced colorectal tumorigenesis.

## Factors affecting microbiota and dysbiosis in IBD

Dysbiosis, an alteration in the gut microbiota, is influenced by numerous factors, including diet, age, genetics, peristalsis, immune responses, antibiotics, nonantibiotic drugs to treat diabetes, the mode of delivery at birth, the method of infant feeding, stress, psychological situations, exercise, and geographical location [54–58]. Further, ethnicity, living with pets (dogs and cats), alcoholism, recreational drugs, environmental pollution, food additives, smoking, and extraintestinal diseases also influence gut microbiota [59]. In addition, any alterations in intestinal defense mechanisms, including the mucus layer, IgA, AMPs, and microRNA (as discussed above), may also contribute to dysbiosis. It can compromise intestinal mucosa integrity and permeability, triggering MALT (Mucosal Associated Lymphatic Tissue) activation, inflammation (leukocytes, cytokines, TNF-α), and tissue damage [60].

Dysbiosis is linked to diseases like type 2 diabetes, allergies, fatty liver disease, obesity, and IBD [61]. For example, *Roseburia intestinalis* and *Faecalibacterium prausnitzii* were found to be lower, while *Lactobacillus gasseri*, S*treptococcus mutans*, Proteobacteria, and certain Clostridiales were higher in individuals with type II diabetes. An increase in *Lactobacillales*, mainly *Streptococcus* species, and a decrease in species belonging to *Bacteroides*, *Eubacterium*, and *Clostridium* were reported in individuals with higher HbA1C (glycated hemoglobin) [57]. In CRC, a handful of bacterial species have been highly implicated due to their production of genotoxic substances [62]. For instance, genetic analysis of the microbiomes collected from colorectal carcinomas continuously reveals elevated levels of *F. nucleatum*, *F. mortiferum*, and *F. necrophorum* within both primary tumor sites and metastasis [63]. It has been postulated that the *Fusobacterium* species contributes to CRC progression by utilizing a galactose adhesion hemagglutinin molecule known as Fusobacterial Fap2 that then binds galactose-*N*-acetyl-*d*-galactosamine within tumor cells [64]. Upon infiltration of tumor cells, Fusobacteria utilize their virulence factor, FadA, to support CRC growth. *Fusobacterium* species also indirectly contribute to the pathogenesis of CRC due to their linkage with known risk factors, namely IBD [63].

**Figure 3:**
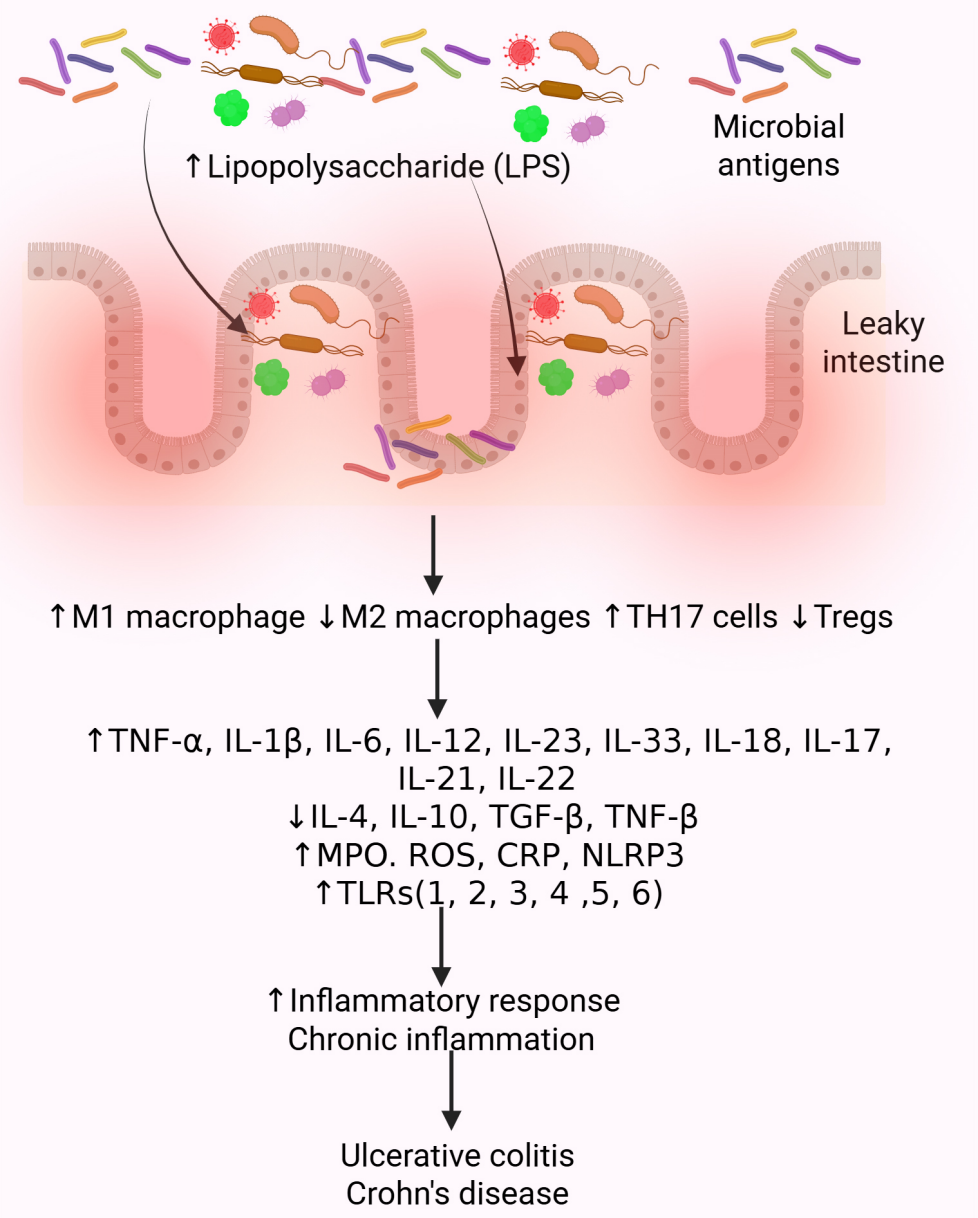
**Molecular changes in intestinal dysbiosis contributing to IBD.** Gut dysbiosis, an imbalance in the gut microbiota, significantly increases the risk of developing IBD, which is characterized by chronic inflammation in the gut. This imbalance can lead to a breakdown of the intestinal barrier, allowing harmful bacteria to penetrate the lining and trigger inflammation. Inflammation is mediated by increased recruitment of immune cells secreting pro-inflammatory cytokines. A decrease in anti-inflammatory cytokines and growth factors, an increase in oxidative stress, and activation of inflammatory mediators such as TLRs contribute to the chronicity of inflammation and the development of IBD. CRP: C-reactive protein; IBD: inflammatory bowel disease; IL: interleukin; MPO: myeloperoxidase; NLRP3: NOD-, LRR-, and pyrin domain-containing protein 3; ROS: reactive oxygen species; TGF: transforming growth factor; TLRs: toll-like receptors; TNF: tumor necrosis factor; Treg: regulatory T. Created in BioRender. Rai, V. (2025) https://BioRender.com/lumhuep

The pathogenesis of IBD involves microbiota changes, immune responses, genetic factors, environmental factors, surgery, lifestyle, hygiene, diet, and drugs [65–67]. A Western-style diet rich in fats and refined sugars can increase ROS (reactive oxygen species) production, inflammation, and dysbiosis via fewer Firmicutes and increased Enterobacteriaceae (dysbiosis) [68]. Crohn’s disease patients specifically show reduced *Faecalibacterium prausnitzii* (Firmicutes), crucial for butyric acid production [69]. Intestinal dysbiosis, at a molecular level, leads to an altered production of microbial metabolites such as SCFAs, LPS, and bile acids, disrupting intestinal barrier function, impacting host physiology, and inducing inflammation. Transgenic mouse models reveal that essential SCFAs such as acetate and butyrate help maintain intestinal homeostasis via signaling through G protein-coupled receptors such as GPR43 that then initiate signal cascades [70]. Ultimately, intestinal barrier disruption causes increased intestinal permeability due to decreased expression of tight junction proteins (leaky intestine) (Figure 3). Increased LPS activates immune cells like macrophages and DCs, involving TLRs, contributing to increased secretion of pro-inflammatory cytokines. Dysbiosis may also contribute to altered gene expression through epigenetic modification involving methylation and histone modification, potentially altering the host response to microbial signals [19, 71–74] (Figure 3).

## Gut microbiota and colorectal cancer

The gut microbiota’s role in the progression from adenoma to carcinoma has been suggested, with a healthy microbiota correlating with a lower risk of advanced adenoma [75]. Factors such as obesity, high-fat diets, smoking, and alcohol consumption, which affect the gut microbiota, are also linked to colon carcinogenesis [76]. As compared to healthy individuals, CRC patients show reduced bacterial diversity and abundance, particularly of *Firmicutes* and *Bacteroidetes* [77]. Gut microbiota influences CRC. The gut microbiota of patients with CRC differs from healthy subjects’ microbiota as they have a lower abundance of commensal bacteria and a higher abundance of carcinogenic bacteria [78]. Certain *Bacteroides* spp. correlates with systemic inflammation and tumor stage [79]. The three most common bacteria seen in CRC patients are *F. nucleatum*, *E. coli*, and *B. fragilis* [78]. *Streptococcus gallolyticus* and enterotoxigenic *B. fragilis* (ETBF) are associated with CRC onset and progression [80].

*F. nucleatum* is typically part of the oral microbiota; however, in CRC patients, it is found in the gut [81]. *F. nucleatum* is correlated with age, tumor diameter, and poor prognosis in metastatic CRC, making it a potential biomarker for proximal colon cancer prognosis. *F. nucleatum* not only correlates with clinical features but also actively modulates the tumor microenvironment by interfering with host immune responses [82]. *F. nucleatum* expression was related to MSI and *BRAF* mutation and was detected in 56% of malignant cases compared with benign lesions. The presence of *F. nucleatum* is also linked to increased infiltration of TIGIT-positive immune cells within the tumor, which are often functionally suppressed, contributing to an immunosuppressive microenvironment compared with benign lesions [79]. *F. nucleatum* can inhibit natural killer (NK) cells and cytotoxic T lymphocytes (CTLs), essential for tumor cell elimination. This is mediated by the Fap2 protein on the bacterial surface, binding to the T cell immunoglobulin and ITIM domain (TIGIT) receptor on NK cells and some T cells. Engagement of TIGIT by Fap2 leads to the recruitment of phosphatases such as SHIP1, resulting in the suppression of PI3K/Akt signaling pathways necessary for NK cell activation and cytotoxicity. As a result, the ability of these immune cells to recognize and destroy tumor cells is markedly reduced. Additionally, *F. nucleatum* can interact with other inhibitory receptors, such as CEACAM1 and Siglec-7, further dampening anti-tumor immune responses [79]. The colon flora is predominantly *E. coli*, and when it spreads to other regions of the gut, it can lead to diseases such as IBD, a contributing factor to CRC. Enteropathogenic *E. coli* (EPEC) is commonly found in CRC patients, and this bacterium suppresses the expression of MMR, a DNA mismatch repair protein, contributing to the development of CRC [83]. *B. fragilis* makes up a very small percentage of total gut microbiota; however, an increase in this bacterium has been seen in IBD, such as Crohn’s [84]. *B. fragilis* releases a toxin that causes inflammatory diarrhea and inflammation-related tumorigenesis [79].

*Streptococcus gallolyticus* has tumor-promoting effects on colon cells, increasing levels of β-catenin, c-Myc, and PCNA, which are associated with cancer development [85]. The higher levels of this bacterium in CRC patients are sustained by CRC-specific conditions such as increased bile acids. ETBF, often linked to diarrhea, peritonitis, and sepsis, has a positive correlation with active IBD and CRC [86]. Co-colonization of toxigenic *E. coli* and ETBF in mice leads to increased IL-17 production and DNA damage, accelerating CRC development [87]. ETBF’s toxin can induce c-myc expression, IL-8 secretion, and STAT3/Th17 immune response activation, further increasing CRC risk [86].

## Dysbiosis and metabolites as biomarkers

Emerging evidence suggests gut microbiota-derived metabolic profiles can provide predictive insights on CRC development. However, a challenge arises in interpreting whether these findings are causative, consequential, or incidental to CRC progression. Serum metabolites analysis in CRC and adenoma patients, 885 serum metabolites were identified to be significantly altered, with 8 gut microbiome-associated metabolites [88]. The study reported a significantly increased *B. fragilis*, *F. nucleatum*, *Parvimonas micra* (*P. micra*), and *Campylobacter jejuni* and downregulation of *Bifidobacterium longum* (*B. longum*) in colorectal patients. Out of 855 colorectal abnormal correlated metabolites, 322 metabolite features exhibited a significant association with the gut microbiome, including *F. nucleatum*, *P. micra*, *Alistipes finegoldii*, *Odoribacter splanchnicus*, *B. longum*, and *Parabacteroides distasonis* [88]. While this study illustrates potential microbe-metabolite associations, it remains correlative, and the temporal dynamics of these changes remain unclear.

A cross-sectional, observational, multi-omics study investigated the temporal dynamics of the metabolome of patients with early-onset CRC (EO-CRC) and late-onset CRC (LO-CRC) [89]. In this study, a comparative metabolic and metagenomic analysis on a discovery cohort comprising 114 EO-CRC patients, 130 LO-CRC, and age-matched healthy controls (97 LO-controls and 100 EO-controls) was performed. Additionally, an independent validation cohort was also included. Both patient groups demonstrated reduced alpha diversity in gut microbiota composition, but EO-CRC patients were observed to have elevated tryptophan, bile acids, and choline production, metabolites with known roles in inflammation and oncogenesis. Conversely, LO-CRC patients demonstrated reduced SCFA production, microbial-produced GABA (gamma-aminobutyric acid) production, and increased acetyl-CoA. While this study demonstrates distinct etiological pathways, causation is yet to be established.

Attempts to distinguish the gut metabolome of colorectal adenoma (CRA) from CRC have also been investigated [90]. In an analysis of fecal and serum metabolites of CRA, CRC, and healthy patients, butyric acid was significantly higher in patients with CRA than in healthy patients. Additionally, healthy patients were enriched with α-linoleic acid and lysophosphatidylcholine, while significantly reduced in patients with CRC. These findings suggest that metabolites may serve as biomarkers for the type and staging of CRC. However, the findings are preliminary, and additional studies with larger, diverse cohorts are required for reproducibility and utility of these biomarkers.

Mechanistic studies may provide insight into the functional consequences of altered microbial metabolites. In the context of CRC, gut metabolites could elicit pro-oncogenic or anti-tumorigenic responses. SBAs derived from microbial transformation of hepatic bile acids are shown to be pro-oncogenic by reshaping the gut microbiota, reducing microbial diversity, suppressing CD8 T cell function, and dysregulating the Wnt pathways implicated in cell proliferation. SFCA, such as butyrate, produced by dietary fiber fermentation, supports colonocyte energy metabolism, maintains luminal hypoxia, and regulates epithelial homeostasis [91–95]. SCFAs reduce the expression of various genes involved in cell proliferation and growth in a human colorectal cell line [96]. CRCs with MSI, when exposed to SCFA, are primed to induce greater CD8+ T cell activation and enhance antitumor activity [97]. Gut microbiota-produced indole derivatives have also been shown to provide protective effects against CRC. Indole-3-lactic acid derived from *Lactobacillus plantarum* has been shown to ameliorate tumor growth, intestinal inflammation, and gut dysbiosis by improving tumor-infiltrating CD8+ T cell activity by decreasing their cholesterol levels through the downregulation of Serum amyloid A3 (*Saa3)*, a key gene involved in cholesterol metabolism [98].

These studies provide a valuable framework for future research to develop clinically useful diagnostic markers. Clinical translation of these diagnostic markers would require integrative, longitudinal studies that incorporate mechanistic validation, host-microbe interactions, and multi-omics approaches.

## Targeting microbiota in IBD

### Microbiota and tumor treatment

Modulation of the gut microbiota and gut microbiota-derived metabolites has been shown to augment antitumor treatment. In a mouse study, phage-induced inhibition of tumor-associated gut microbe *F. nucleatum* enhanced the efficacy of first-line chemotherapy drugs [99]. Conversely, altered microbiota due to chemotherapy may also confer resistance to chemotherapeutic drugs. In a longitudinal study, 16S rRNA sequencing on 353 fecal specimens exhibited reduced microbial diversity after neoadjuvant chemoradiotherapy (nCRT) [100]. The investigators found that nCRT-induced enrichment of *Bacteroides vulgatus* (*B. vulgatus*), leading to *B. vulgatus*-mediated nucleotide biosynthesis, protected cancer cells with upregulated DNA repair and nucleoside transport genes. These findings were confirmed in the same study when nucleoside supplementation or *B. vulgatus* gavage protects cancer cells from the 5-fluorouracil or irradiation treatment [100].

Metabolites of microbiota, for example, indole-3-carboxaldehyde (I3A) and indole-3-propionic acid (IPA), have been proven in their abilities to reduce inflammation, support recovery of blood-forming organs, decrease bone marrow suppression, enhance GI function, and maintain the structure of the GI tract, even after radiation exposure [101]. In the same way, SCFAs produced by microbiota act against radiotherapy in the restoration of the epithelial barrier and overall gut integrity through mechanisms of, for example, HIF-1 expression and regulation of tight junctions, along with increased mucin production [102]. Conversely, dysbiosis of gut microbiota can worsen radiation enteritis by inducing epithelial inflammation and the malfunction of the barrier and increasing the expression of pro-inflammatory cytokines such as TNF-α and IL-1β [103].

Also, the modulation of gut microbiota affects the responses to treatments that are related to immune actions. The gut flora shapes the host immune responses in the tumor microenvironment, which is mediated by both innate and adaptive immune mechanisms and influences the effects of the immune checkpoint inhibitors, including PD-1/PD-L1 blockers. For example, the antibiotic-treated patients demonstrate less effect from PD-1 blockers; however, this insensitivity could be reversed by fecal flora transplantation [104]. *F. nucleatum* upregulates the succinate production that induces tumor cell immune resistance against anti-PD-1 monoclonal antibody treatment, while its elimination resensitizes tumor cells to this treatment [105]. Other interventions that render soluble fiber pectin as a probiotic food source have been shown to increase the efficacy of anti-PD-1 therapy under the preclinical setting through finding increased secretions of the metabolite indole-3-carboxylic acid by *Lactobacillus gallinarum* [106, 107]. Changes in gut bacteria and their by-products have been proven to increase the effectiveness of the treatment in several ways. Some bacteria, for instance, *Salmonella typhimurium*, can enter and grow in tumors because these tumors create a weak defense area around them. These bacteria then kill the tumor cells by destroying blood vessels through TNF-α, and also activate immunity signaling in the tumor cell to stimulate macrophage phagocytosis and autophagy in the tumor cell itself [108]. In mice, making a certain group of tumor-promoting bacteria from the gut, *F. nucleatum*, less active with viruses increased the effect of the common chemotherapy drugs. However, treatment-related changes in the flora may sometimes result in bacterial resistance against the medications. For example, the increase of an ordinary gut bug such as *B. vulgatus* leads to the increased synthesis of nucleotides after the combined radiation and medicament therapy before surgery, and this feeds the tumor cell with more DNA-repair and nucleoside-transporting genes. Further, nucleoside feeding or feeding of *B. vulgatus* protects cancer cells against irradiation with 5-FU. Also, *F. nucleatum* may help chemoresistance by targeting TLR4 and MYD88 (myeloid differentiation primary response 88) innate immune signaling, and some specific microRNAs to activate the autophagy pathway. Thereby reducing apoptosis and altering the response of CRC cells to chemotherapy [109].

Apart from this, bacterial CpG-ODN can cause an innate immune response resulting in TNF-α driven hemorrhagic necrosis and also leading to adaptive immunity wherein both CD8+ and CD4+ T cells cooperate to eliminate tumors. For instance, the BCG (bacille Calmette-Guerin) vaccine works against tumors by using CD4 T cells and interferon-γ signaling, not via MHC-II (major histocompatibility complex class II) restriction [110]. Altogether, these findings highlight the dual role of the gut microbiota in cancer therapy, where specific bacterial species and their metabolites can either hinder or enhance the efficacy of conventional and emerging treatments. Targeted microbiota manipulation through phage therapy, antibiotics, fecal transplants, or diets is a hopeful plan to fight tumors and improve patient outcomes.

### Improving microbiota while on colorectal cancer therapies

Targeting microbiota to improve treatment for CRC is still a novel strategy. Treatment for CRC depends on multiple factors, including the cancer stage and the age of the patient. Current treatment for CRC typically involves chemotherapy, surgery, radiotherapy, immunotherapy, and other modalities [111]. Non-metastatic CRC is typically resected with surgery followed by chemotherapy. Surgery is seen as a curative measure; however, 35% of patients will have the cancer recur and eventually metastasize [112]. At initial diagnosis, CRC has metastasized in 22% of patients [113]. For metastatic CRC, treatment typically involves chemotherapeutic drugs of 5-fluorouracil, capecitabine, irinotecan, and oxaliplatin, along with folinic acid. Unfortunately, chemotherapy can lead to more problems, as CRC cells can become resistant to it [5]. Because of the interaction of microbiota and CRC, therapies involving it have gained more traction. Current therapies for CRC, including chemotherapy and radiotherapy, have been proven to affect microbiota as they reduce the diversity of the microbiota, which can affect patient outcomes to treatment [114]. However, currently, there are no therapies to improve microbiota while on anticancer treatment. A clinical trial, focusing on prebiotics, is exploring the effect of xylooligosaccharides (XOS) on CRC patients receiving chemotherapy. The aim is to assess whether prebiotics can increase the bioavailability of cytotoxic drugs, reduce adverse effects, and improve the quality of life by modifying gut microbiota. XOS feeds beneficial bacteria such as *bifidobacteria* and *lactobacilli* within the digestive tract [115]. Currently, the potential of FMT (fecal microbial transplant) is being discussed, but it has not been utilized for CRC patients.

The microbiota is a complex, diverse community unique to every person. It consists of both good and bad bacteria, protists, and fungi. Because it has such an intimate relationship with its host, a rise in certain bacteria can lead to detrimental effects. It has been shown that patients with CRC have dysbiosis, and finding solutions to improve it can lead to better outcomes. Finding the microbiota is uniquely diverse, and finding the correct bacteria that will benefit the patient without causing more adverse effects is difficult. Introducing prebiotics to a patient undergoing chemotherapy may lead to a risk of infection as chemotherapy weakens the immune system. Fecal matter transplantation theoretically shows potential, but more studies will have to be done to show its effectiveness for CRC patients. Currently, plenty of data shows the potential of utilizing microbiota to improve CRC outcomes. However, it is still early in its development, and clinical research has just started.

#### Probiotics/Prebiotics

Probiotics can modulate host microbiomes due to their presence within the GI tract. Previous studies supported that postoperatively, probiotics successfully decreased rates of infection [116]. Yet, the role played by probiotics in the process of oncogenesis remains debatable in terms of its benefits and risks. Franko et al. [116] investigated the interplay between the microbiome and CRC therapy in a double-blind trial with short-course perioperative oral probiotics in 120 patients undergoing major GI surgery. In the study, 57 patients were prescribed oral probiotic VSL #3 (a probiotic consisting of strains of *Bifidobacteria*, *Lactobacillus*, and *Streptococcus*), and 63 patients, a placebo [116]. The probiotic and placebo were dispensed simultaneously in both groups, with patients receiving one dose preoperatively and two doses per day postoperatively until being discharged, with a maximum of 15 doses. After a follow-up of six years, the results showed no statistical difference (*p* = 0.317) in survival between the two groups, suggesting that probiotics did not make any difference in the survival rate of patients diagnosed with curable CRC. These results suggest that probiotics do not have systemic anti-cancer effects, although they have effects at the local microbiome level [116]. Data from mouse models support such conclusions by showing that certain strains have benefits. For instance, *Lactobacillus* JY300-8 and JMR-01 reduced the incidence of CT26 tumors to between 13% and 17% in both the live and inactivated forms, indicating that there are direct anti-tumor effects irrespective of the viability of the cells [117]. In this study, CRC was induced in the CT26 cells of mice that received the probiotics and those that didn’t, i.e., the control group. Another layer of complexity was added in that the two probiotics were administered with either living or inactivated bacteria. While the control group developed tumors at a rate of 97%, the mice given living and inactivated bacteria only developed tumors at a rate of 13% and 17%, respectively [117]. These outcomes strongly support the administration of probiotics containing *Lactobacillus* to reduce tumorigenesis in CRC. Clinically, such probiotics as Colon Dophilus alleviate chemotherapy-induced diarrhea and enterocolitis, hence indirectly giving them a possibility of making the treatment tolerable [118].

Prebiotics such as Simbio-flora also showed promising results in CRC patients who underwent tumor resection. Prebiotics like Simbio-flora, which enrich *Lactobacilli* and *Bifidobacterium lactis* HN019, shorten hospital stay and reduce postoperative inflammation in CRC patients [119]. While these diverse outcomes underscore the many benefits that both prebiotics and probiotics possess in the treatment of CRC, further investigation and validation of strain and dose are warranted [120].

#### Fecal transplant

FMT acts as a strategy to ameliorate dysbiosis and increase CRC therapy. The Chinese herbal remedy known as Xiao-Chai-Hu-Tang (XCHT) has traditionally been used to treat depressive symptoms, which are known to contribute to tumorigenesis [121]. In this study, xenograft mice under chronic stress and treated with XCHT [includes Bupleurum root (Chai Hu), Pinellia tuber (Ban Xia), Scutellaria root (Huang Qin), Ginseng (Ren Shen), Jujube (Da Zao), Licorice (Gan Cao), and Ginger (Sheng Jiang)] reduce the tumor volume by 60%. The underlying mechanism was inhibition of antiapoptotic proteins Bcl-2/Bcl-xL [121]. This was attributable to the fact that the FMTs containing XCHT increased the concentration of various LPS-producing bacteria, including *Desulfovibrio*. FMT can also transfer antitumor immune cells that are enriched with *B. vulgatus*, increased CD8+ cells, and NK cells in murine CRC models with an elevation of TNF-α and IFN-γ levels [122]*.* Translating these results in humans is important; however, variations between mouse models and humans due to differences in the microbiota are of concern. The host-donor response hinted by preclinical data to be ideal in the acquisition of beneficence is hindered by the change in microbiota and undermines human real-life application [123]. The earliest trials uncovered that it may have a potential application in the attenuation of colitis secondary to immune checkpoint inhibitors. However, larger studies are needed to determine the role of this therapy in the primary treatment of CRC [124].

#### Diet

The gut microbiome plays an essential role in host metabolism as bacteria break down various non-digestible compounds. Improper diet can therefore contribute to poor health outcomes due to changes in the intestinal mucosa and increased inflammation. The consumption of large amounts of processed red meats contributes to CRC due to the production of *N*-nitroso compounds that disrupt the intestinal lining [125]. Similarly, high-fat diets increase the concentration of lysophosphatidic acid, thus disrupting normal metabolism and contributing to tumorigenesis [126]. Meanwhile, large amounts of fiber help increase the concentration of bacterial species such as *Bifidobacteria* and *Lactobacillus*, leading to increased butyrate levels that help suppress tumor growth [125]. The role of these two species has been documented in previous studies in which the fecal microbiota of healthy participants was analyzed via fluorescent in situ hybridization [127]. Participants were instructed to consume First Leaf (blackcurrant extract powder, lactoferrin, and lutein) and Cassis Anthomix 30 (blackcurrant extract powder; either 672 mg/day or 1,500 mg/day), which both contained blackcurrant extract powder, while the former also contained lactoferrin and lutein. Interestingly, the results equated the two compounds to probiotics as they each helped increase the concentration of beneficial bacteria, including *Lactobacilli* and *Bifidobacteria* [127]. If studies such as this solidify the relevance of a healthy diet for its preventative nature, then one could similarly question its positive impact throughout CRC treatment. This has been the case for patients prescribed the chemotherapeutic drug 5-fluorouracil when administered in concert with vitamin B6 and pyrimidines. When the two nutrients are consumed in large quantities, they disrupt bacterial metabolism of folate, thus altering folate metabolism and the effects of 5-fluorouracil. Similarly, diet can alter the host microbiota in patients administered cytostatic drugs like fluoropyrimidines during their treatment of CRC [125].

The benefits that come with incorporating a healthy diet during treatment are profound and should be maintained even if patients are declared cancer-free. The findings of randomized clinical trials involving patients previously diagnosed with adenomas prove that calcium supplementation decreased rates of adenoma recurrence [128]. The trials proved that supplementation results in increased calcium levels within extracellular spaces, which then activate calcium-sensing receptors responsible for mediating interactions between microbiota and immune responses [128]. Given the rich source of calcium within milk, studies have revealed that other dairy products, such as conjugated linoleic acid, reduce carcinogenesis [129]. However, the relationship between food and cancer is not so clear-cut; for instance, evidence shows that consumption of large amounts of fermented milk products can negatively alter gut microbiota and propel CRC [128]. Even though this echoes a theme that defining certain foods as purely protective or harmful is tricky, there exists a consensus that the following are protective: milk, cheese, fruits, and whole grains, amongst others, including moderate amounts of wine. In contrast, high-risk foods include red meat, processed meat, high fructose corn syrup, animal-derived fat, and high levels of alcohol consumption [130]. The research regarding diet and tumorigenesis is still incomplete, and conscious dieting possesses great potential for the future of CRC prevention.

#### Antibiotics

A patient’s microbiota can be improved throughout their treatment of CRC by selectively removing harmful species. One of the key bacterial species associated with CRC is *Fusobacterium*, while *Bacteroides*, *Selenomonas*, and *Prevotella* also prevail with metastasis. Previous studies on xenografted mice show that treatment with the antibiotic metronidazole was shown to successfully reduce the concentration of *Fusobacterium*, which had the additional effect of minimizing cancer cell proliferation as well as tumor growth [131]. When administered preoperatively, antibiotics were shown to enhance CRC patient survival by roughly 25% [132]. With regards to mouse models, the formulation and administration of an antibiotic known as silver-tinidazole encapsulated in liposomes (LipoAgTNZ) helped eliminate tumor-associated bacteria [132]. More specifically, the antibiotic reduced the *F. nucleatum* load as well as *E. coli* Nissle spp., all without causing dysbiosis. By targeting *F. nucleatum*, LipoAgTNZ enhanced survival rates by 70% within two different CRC models, thus underscoring the benefits that antibiotics possess for a host’s microbiota throughout his or her treatment.

## Conclusions

The intricate interplay between gut microbiota and CRC underscores a complex and evolving landscape of disease mechanisms and potential therapeutic strategies. Dysbiosis, marked by alterations in microbial composition and function, has been implicated in CRC pathogenesis through its effects on immune responses, mucosal integrity, and microbial metabolite profiles. The gut microbiota’s role in CRC progression highlights both harmful and beneficial aspects, with certain bacteria promoting inflammation and tumorigenesis, while others exhibit protective effects through metabolite production. Emerging evidence suggests that gut microbiota can serve as valuable biomarkers for CRC detection and progression, with specific metabolites and microbial profiles offering insights into disease stages and therapeutic responses. The modulation of gut microbiota, through interventions such as probiotics, prebiotics, and dietary changes, presents a promising avenue for enhancing CRC treatment outcomes. As research advances, a more nuanced understanding of microbial interactions and their impact on treatment efficacy will be crucial for developing personalized and effective therapeutic approaches. Future studies should continue to explore the mechanistic pathways linking dysbiosis with CRC, aiming to harness the microbiota for innovative treatment strategies and improved patient outcomes.

## Abbreviations

AMPs: antimicrobial peptides
CRA: colorectal adenoma
CRC: colorectal cancer
DCs: dendritic cells
DEFA5: α defensin 5
EO-CRC: early-onset colorectal cancer
ETBF: enterotoxigenic *B. fragilis*
FASN: fatty acid synthase
*FBXW7*: F-box and WD repeat domain containing 7
FMT: fecal microbial transplant
GI: gastrointestinal
HBD1: human β-Defensin 1
IBD: inflammatory bowel disease
IFN: interferon
IL: interleukin
LipoAgTNZ: silver-tinidazole encapsulated in liposomes
LO-CRC: late-onset colorectal cancer
LPS: lipopolysaccharide
MMP: matrix metalloproteinase
MSI: microsatellite instability
Mzb1: marginal zone and B1 cell-specific protein
nCRT: neoadjuvant chemoradiotherapy
NK: natural killer
NOD2: nucleotide-binding oligomerization domain 2
SBAs: secondary bile acids
SCFAs: short-chain fatty acids
sIgA: secretory IgA
STAT3: signal transducer and activator of transcription 3
TH1: T helper 1
TLRs: Toll-like receptors
TNF: tumor necrosis factor
Treg: regulatory T
UC: ulcerative colitis
XCHT: Xiao-Chai-Hu-Tang
XOS: xylooligosaccharides

## Declarations

### Copyright

© The Author(s) 2025.
